# Active children through incentive vouchers – evaluation (ACTIVE): a mixed-method feasibility study

**DOI:** 10.1186/s12889-016-3381-6

**Published:** 2016-08-26

**Authors:** Danielle Christian, Charlotte Todd, Rebecca Hill, Jaynie Rance, Kelly Mackintosh, Gareth Stratton, Sinead Brophy

**Affiliations:** 1 Swansea University Medical School, Swansea University, Swansea, SA2 8PP UK; 2 College of Health and Human Science, Swansea University, Swansea, SA2 8PP UK; 3 College of Engineering, Bay Campus, Swansea University, Swansea, SA1 8EN UK

**Keywords:** Adolescent, Teenager, Physical activity, Accelerometer, Voucher, Deprived, Mixed methods

## Abstract

**Background:**

Adolescents face many barriers to physical activity, demonstrated by the decline in physical activity levels in teenage populations. This study aimed to assess the feasibility of overcoming such barriers via the implementation of an activity-promoting voucher scheme to teenagers in deprived areas.

**Methods:**

All Year 9 pupils (*n =* 115; 13.3 ± 0.48 years; 51 % boys) from one secondary school in Wales (UK) participated. Participants received £25 of activity vouchers every month for six months for physical activity or sporting equipment. Focus groups (*n =* 7), with 43 pupils, and qualitative interviews with teachers (*n =* 2) were conducted to assess feasibility, in addition to a process evaluation utilising the RE-AIM framework. Quantitative outcomes at baseline, five months (during intervention) and twelve months (follow-up) included: physical activity (accelerometer), aerobic fitness (12 min Cooper run) and self-reported activity (PAQ-A). Motivation to exercise (BREQ-2) was measured three months post-baseline and at follow-up.

**Results:**

Qualitative findings showed that vouchers encouraged friends to socialise through activity, provided opportunities to access local activities that pupils normally could not afford, and engaged both those interested and disinterested in physical education. Improvements in weekend moderate-to-vigorous physical activity and reductions in sedentary behaviour were observed in both sexes. Boys’ fitness significantly improved during the voucher scheme. ‘Non-active’ pupils (those not meeting recommended guidelines of 60 mins∙day^−1^) and those with higher motivation to exercise had higher voucher use.

**Conclusions:**

Adolescents, teachers and activity providers supported the voucher scheme and felt the vouchers enabled deprived adolescents to access more physical activity opportunities. Voucher usage was associated with improved attitudes to physical activity, increased socialisation with friends and improved fitness and physical activity; presenting interesting avenues for further exploration in a larger intervention trial.

**Electronic supplementary material:**

The online version of this article (doi:10.1186/s12889-016-3381-6) contains supplementary material, which is available to authorized users.

## Background

Physical inactivity in youth has been linked to a number of health issues in later life [1] and is considered a key contributor to increases in obesity [2]. This is concerning since a notable decline in physical activity (PA) is observed during adolescence [3] and lower PA levels, fitness and higher cardiometabolic risk scores are reported in children in deprived areas [4–7]. Therefore, approaches to address physical inactivity in this target group are urgently required.

The main barriers to PA for adolescents are reported to be cost, accessibility, lack of parental support and lack of local facilities [8–10]. The Households Below Average Income data (HBAI) for the United Kingdom (UK) showed that the most disadvantaged families were unable to purchase leisure equipment for their children, such as sports kits or bicycles, due to prohibitive costs, and nearly a quarter of disadvantaged families reported a lack of outdoor space or facilities for their children to play safely [11].

Participating in organised activities more than once a week is associated with improved fitness [12]. However, participation is often lower for children in deprived areas and children with inactive parents [13]. Additionally, there is a difference between what young people would like to do and what is available [14], meaning they may not have the opportunity to participate in desired activities. For example, research has reported that girls often see competitive sports as a barrier to participation [15]. Therefore, if the majority of activities available are competitive, there is likely to be a lower uptake and lower physical activity among girls. This study examines whether placing decision making with young people, through activity vouchers, can increase empowerment and engagement to shape activity provision.

Previous research has reported positive effects on PA levels following provision of activity vouchers to adults in socially deprived communities [16, 17]. However, the extent to which behaviour change can be sustained following such schemes remains uncertain [16, 18]. Furthermore, vouchers were used as an adjunct to motivational interviewing or provision of PA information in these studies. Provision of PA vouchers as a stand-alone intervention has not been evaluated among adolescents.

Recent research among primary school children reported a positive increase in the percentage of time spent using active transport following the incentive of entry into a cash or voucher prize draw for utilising active transport every weekday [19]. Increases in fitness centre use have also been found among college students following monetary incentives [20]. However, these two previous studies [19, 20] focused on financial reward for participation rather than direct provision of activity enabling vouchers.

This study aimed to conduct a phase 1 feasibility study of the ACTIVE (Active Children through Incentive Vouchers - Evaluation) scheme among adolescents in a deprived community, in order to assess acceptability of the study design and explore the effects of physical activity, fitness and motivation to exercise.

## Methods

### Recruitment

One secondary school in a deprived catchment area in South Wales (UK) was approached to take part in the scheme. The school was classified as deprived as it: i) reported 54 % eligibility for free school meals (FSM) [21]; ii) was eligible for ‘Community Regeneration Initiatives’, ‘Communities First Funding’ and ‘Objective One’ priority areas; and iii) is in one of the more socially deprived areas in Wales [22]. All Year 9 pupils (13.3 ± 0.48 years) were eligible to receive activity vouchers (*n =* 115, 59 boys; 49 eligible for FSM).

### Intervention

The intervention involved provision of £25 of vouchers (five vouchers in increments of £5) per month for six months. Vouchers could be used to: i) enrol in existing activities; ii) fund coaches or new activities directly in communities or at their school, such as Zumba and Boxercise and iii) purchase new sporting equipment for themselves or their school. Recognised providers (i.e., leisure centres, clubs, and dance providers) were recruited during development stages, and their logos were printed on the vouchers to enable easy identification of where they could be used.

Vouchers were numbered and contained the participant’s name with a section to complete the type and duration of activity and the signature of the participant and provider. Vouchers were treated like a cash transaction, but no change was provided in order to prevent non-PA-related purchases. At the end of the month, all vouchers were collected from each provider with accompanying invoices for monetary reimbursement.

A trained facilitator regularly attended the school to highlight activities available in the area, provide advice on how best to access activities, and discuss methods of overcoming individual barriers to exercise. The facilitator liaised between local sporting providers and pupils to ensure vouchers were redeemable when accessing facilities and identify new coaches or facilities.

### Outcomes

#### Qualitative outcomes

**Fig. 1:**
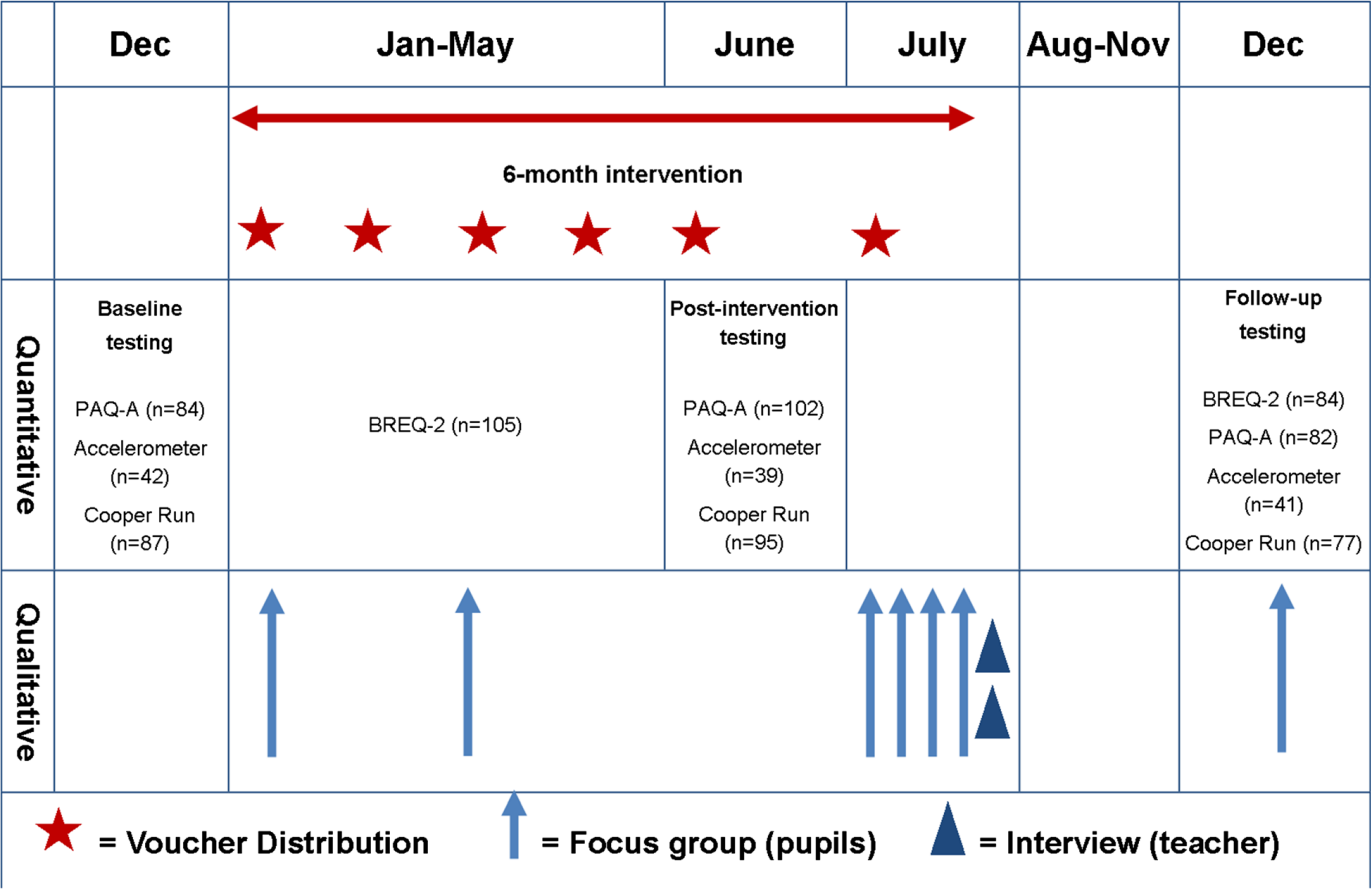
ACTIVE intervention timeline

Prior to the intervention, one focus group was conducted with Year 9 pupils (*n =* 10, 5 boys) to examine how best to introduce the scheme, establish preferred PA providers, and identify potential barriers to participation (Fig. 1). Pupils were purposively allocated to focus groups depending on sex and deprivation, but were then selected randomly to participate from these groups by selecting every 10th pupil from the separate lists derived from the purposive sampling. Deprivation was classified at an individual level through FSM eligibility [21].

**Fig. 2:**
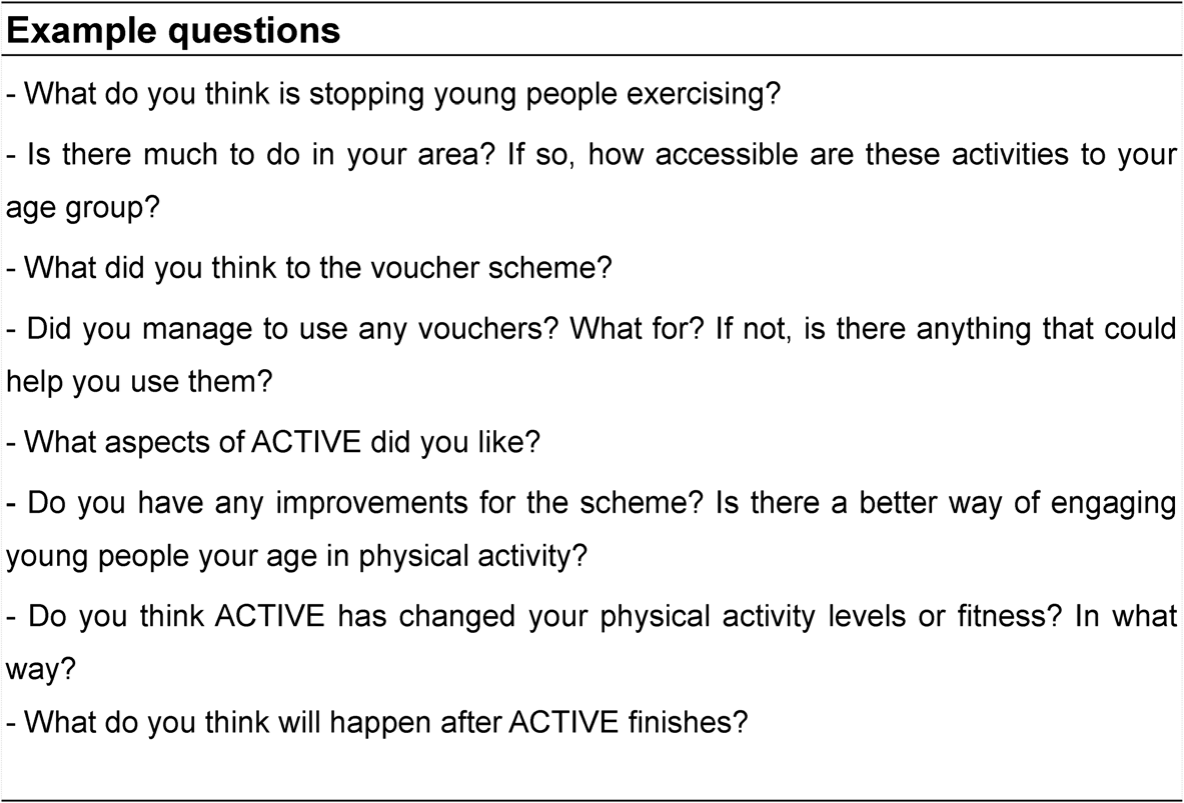
Example of topic guide for focus groups and interviews

Two experienced researchers were present during focus groups (DC & SB or DC & RH), which took place in an empty classroom and lasted one hour on average. Each session was digitally-recorded following consent from pupils and parents, for later transcription, and followed a semi-structured topic guide (Fig. 2). No *a priori* hypothesis was determined, and themes and codes emerged through data analysis following transcription. All subsequent focus groups followed the same methodology with five to six pupils present at any one time [23].

A second focus group was conducted mid-intervention with five adolescents who were not using their vouchers, in order to identify barriers to use and suggest changes to improve uptake. Additionally, four focus groups (two girl groups (*n =* 11) and two boy groups (*n =* 12)) and two interviews with school teachers (Head of Year 9 and Head of P.E.) were conducted post-intervention. These interviews followed a similar line of questioning to that of the pupils (Fig. 2) but mainly concentrated on the teachers’ views of the scheme and whether they had perceived any changes in their pupils’ behaviour as a result of ACTIVE. A final focus group (*n =* 5) was conducted during the six month follow-up to examine longer-term changes in PA.

#### Quantitative outcomes

Quantitative outcomes included objectively measured PA, self-reported PA and aerobic fitness. More details can be found in Additional file 1.

### Data analysis

Thematic analysis was used to analyse qualitative data. Focus group and interview transcripts were coded independently by two members of the research team and discrepancies were resolved through discussion. The themes and findings were presented back to participants during follow-up as suggested in the Consolidated Criteria for reporting qualitative research (COREQ) checklist [24].

## Results

The RE-AIM framework was used to evaluate the reach, effectiveness, adoption, implementation and maintenance of the project [25].

### Reach

All 115 pupils in Year 9 were eligible to participate and were provided with ACTIVE vouchers. At least one voucher was used by 81 % (48/59) of boys and 77 % (43/56) of girls. At least one voucher was used by 76 % (37/49) of deprived participants and 83 % (53/64) of non-deprived. Mid-intervention reasons for non-participation were lack of time, lack of opportunity to use vouchers, and apprehension over where vouchers were accepted.

### Efficacy/effectiveness

#### Voucher usage

During ACTIVE, 1464/3450 (42.6 %) of vouchers were used. Boys used more vouchers than girls (807 boys: 657 girls). Girls were more likely to use their vouchers to buy equipment than boys, 61 % of vouchers compared to 42 %, respectively [Difference 18.9 % (95 % CI: 13.8 %–23.8 %)]. Popular uses included purchasing scooter equipment (22.2 % boys, 38.5 % girls), football sessions (16.7 % boys), skateboarding and skateboard equipment (15.4 % boys), rollerblades (11.3 % girls), and attending the local waterpark (21.9 % boys, 13.7 % girls) (Table 1). Vouchers were predominantly used to attend pre-existing activities in groups or to buy equipment, as opposed to funding new coaches. Pupils did suggest activities or new activity providers to the facilitator but did not tend to organise new coaches or activities themselves. There was a preference for voucher expenditure at weekends or during half-terms, averaging 11 vouchers per weekend day and eight vouchers per half-term day. Normal school days showed the least voucher usage with an average of only five vouchers being spent per weekday.

**Table 1:** Frequency data of voucher usage stratified by gender

Activity/Equipment	Total	Girls	Boys
Assault Course	31	31	0
Badminton	5	5	0
Basketball	2	0	2
Basketball Equipment	10	2	8
Boxercise	1	0	1
Boxing	2	0	2
Boxing Equipment	4	0	4
Climbing	9	7	2
Cycling accessories	29	9	20
Football	135	0	135
Football Equipment	2	2	0
Gym (One off session)	37	28	9
Gym (Membership)	18	13	5
Laserzone	135	66	69
Rollerblades	88	74	14
Rugby Equipment	31	12	19
Scooter Equipment	432	253	179
Scootering	24	16	8
Skateboard Equipment	107	34	73
Skateboarding	55	4	51
Surfing Equipment	10	0	10
Swim Equipment	2	2	0
Swimming	23	9	14
Tennis Equipment	1	0	1
Waterpark	267	90	177
Missing data	4	0	4
Total	1464	657	807

#### Evidence of impact on PA and fitness

A brief overview of the evidence of impact will be presented here, but further details can be found in Additional file 1. A marginal increase in moderate-to-vigorous PA (MVPA) was observed during the scheme. However, when stratified by day of the week, weekend MVPA showed a significant increase during the intervention in both sexes. A significant decrease in sedentary behaviour (SB) was reported overall during the intervention, though this was not sustained twelve months post-baseline. Furthermore, the intervention was associated with improvements in fitness post-intervention; significant for boys.

### Adoption

Of the 24 activity providers participating, 16 were utilised (Table 1). Providers not used were those initiated by researchers at baseline and were predominantly structured activities including; dance classes, swimming clubs, gymnastics classes, and football coaching.

### Implementation

The ACTIVE scheme was implemented as intended with pupils receiving vouchers through school teachers. Positive feedback was received from teachers, who reported the scheme was feasible to run through school and was not too intrusive on school time.

### Maintenance

Quantitative data showed promise for sustainability both in terms of engagement and impacts on health behaviours (Additional file 1), though these need to be assessed on a larger scale before accepting conclusiveness. Qualitative responses regarding the scheme are discussed below with pupil and teacher quotes presented in Tables 2 and 3, respectively.

**Table 2:** Qualitative data from pupils regarding positive aspects of the voucher scheme and recommendations for improvement

Positive aspects of the voucher scheme	
Removal of financial barrier	*“…like when you go like with your friends and you don’t have the vouchers you like have got to worry about what you’re gonna pay, who’s got money, who hasn’t, but like this just everyone’s got it and you can just go…” (girl)*
Changed attitudes towards physical activity	*“…Well I didn’t really like sports until I started using the vouchers. I do a bit more sport than I used to…” (girl)* *“…The girls, they do gym and all that like when you goes out, you just sees them all the time…” (boy).* *“…I think it has worked because some people I think have got a gym membership after they went to the gym and when they stopped, when the vouchers stopped, I think they carried on going which they wouldn’t have before we had the vouchers…” (girl)*
Increased choice / flexibility	*“…With the vouchers we can do it anytime… you get more of a choice with the vouchers…” (boy)* *“…That’s why nobody likes P.E. because it forces them to do things they don’t want to do…”(girl)* *“…I like this input, yeah, like get a say in what we’d like…”(girl)* “…*that’s why I chose equipment because when it (the vouchers) finishes you can stop doing things but if you’re broke that much you can still use it…” (girl)* *"It’s cheaper to like pay for activities, like to go swimming and that than for like scooters and that’s more expensive so the voucher would be more better for equipment…"(girl)*
Increased awareness of local facilities	*“…I don’t know, the stuff that was on the back of it, it looked, fair play, it was like you want to go and do it so basically that’s what I went in to do in places and I did stuff. Because they’re on the back you can do it so why not try it out…”(girl)*
Increased social interaction	*“…It builds your friendship more as well from places…because like it’s doing something fun and you also doing it with your friends, like mixing with them…”(boy)* *"It’s made everyone active because the people who sometimes aren’t active might be friends with someone who is active and they have more opportunities to go and do different stuff (laughter) that they haven’t done before with the vouchers. And then they get like the unfit people and drag them along because they’re unfit…"(girl)* *“…you can do exercise in places like I enjoy going to LaserZone, I find it really fun, there for like half an hour and I’m sweating pints so…” (boy)* *“…because we’ve got the money, we’re not on the streets just making trouble, we’re out doing something…” (boy)*
Increased competence / body image	*“…Personally I think it’s helped me because I’ve become more fit and better stamina and things like that but other people in the year… when I was doing it, I had a six pack…” (boy)* *“…Yeah, it makes like unfit people going to be like fit because like they can go swimming and stuff..” (boy)*
Recommendations for improvement	
Provision of an electronic card	*“…Yeah the paper falls apart when its wet…”(boy)* *“…because then sometimes they say it’s like only £3 and you’ve got a fiver then you’ve lost £2. So if you go twice you’ve, say you’ve spent £6 and then you’ve lost £4 what you could have spent on another one, on another sports…” (boy)* *“…Reckon you should like make a little card or something, we don’t have to carry paper around with us everywhere…and say how much we’ve got left on our cards and then use it…” (girl)*
Vouchers redeemable for public transport	*“…I think if we could improve the vouchers they should have like bus passing and that…I think it might get more people active because it’s easier to get places if it’s free to get there, it’s free to do it…”(boy)*
Increased range of opportunities	*“If they had treadmills in school, I’d be happy…Actually treadmills at school, that would keep everyone active…I don’t know if you look like(name of sports centre)…all the equipment they’ve got, just bring some things into school like that and like they could impel the people like to be more active…”(boy)* *“....I was going to buy some weights but I don’t know where I could buy them…”(boy)* *“…I’d like to see like, I’ve been like looking online for like female boxing and stuff like that, they don’t really do that…” (girl)* *“…It’s only like one thing in (name of area) and that’s the(name of activity centre) (laughs) isn’t it basically*…”(girl) *“…Getting all of us just on a trip....Yeah, and go up to (next town) for the day, like spend your vouchers…”(girl)* *“…Everyone’s going to go paintballing, you’d have loads going for paintballing…” (boy)*
Use during school holidays	*“…I would like one of them vouchers just for the summer holidays because you know like when parents are buying the new like school bags and everything and it’s going to be expensive to get everything…and then you’ve got the vouchers you don’t have to ask for money…” (girl)* *“…When you get home you just want to relax, it’s like I’d use them more weekends probably…I’d use them more in the holidays probably…” (girl)*

### Positive aspects of the voucher scheme (pupils)

#### Removal of financial barrier

Participants commented that vouchers enabled them to fund activities or buy equipment not normally accessible to them due to parental monetary constraints (Table 2). Pupils mentioned that prior to the scheme, if one or two adolescents could not afford the activity, the whole group did not go. Vouchers enabled participation by providing everyone the same opportunities regardless of economic background. It was felt that this sense of inclusiveness reduced the stigma linked to deprivation experienced by those unable to participate due to lack of financial resources.

#### Changed attitudes towards PA

Participants noted that their attitudes towards PA changed for the better, as for many the only experiences of activity had been through school P.E. They suggested seeing people from their year group in local activity centres reinforced the acceptability of participating in activities outside school; activity centres became a venue for socialising, and activity was seen as an opportunity to have fun with friends. Pupils remarked that the vouchers stimulated an interest in PA, which continued after the scheme ended, and believed this long term stimulation of interest would not have happened without the scheme.

#### Increased choice/flexibility

The large number of companies supporting the scheme provided flexibility so that one week a pupil could, for example, buy a skateboard, next week join a gym, and a month later, attend the waterpark. The option to spend vouchers on equipment, as well as activity, was praised as this gave financially-disadvantaged families the opportunity to buy longer-lasting, more expensive sporting equipment such as scooters, which were previously unaffordable. Additionally, participants liked that vouchers were not restricted to specific times or days, which would otherwise reduce accessibility.

#### Increased awareness of local facilities

Participants reported an increased awareness of local services and opportunities to try existing PA provisions in their area.

#### Increased social interaction

Some participants commented that the vouchers allowed them to spend more time doing activities with family and friends, which increased their enjoyment. In fact, several noted that those less inclined to take part in activity were now participating as they would go to be sociable and have fun with friends. One boy mentioned that he welcomed the opportunity to do something productive in the evenings, minimising his involvement in anti-social behaviour.

#### Increased competence/body image

The scheme also led to reported increases in competence and improved body image (particularly among boys), possibly due to improved self-efficacy.

### Recommendations for improvement of voucher scheme (pupils)

Most participants were positive regarding the scheme and many were keen for the vouchers to return in the next academic year. However, there were recommendations for improvements (Table 2).

#### Provision of an electronic card

Some participants reported the paper vouchers ripping or disintegrating when wet, so the predominant suggestion was provision of an electronic card, also ensuring the exact activity cost could be deducted, instead of payments being made in increments of £5. Participants suggested they would be less likely to lose an electronic card.

#### Vouchers redeemable for public transport

It was suggested that vouchers should be redeemable for transport or that pupils should have access to free bus passes through the local council to access facilities outside of the community. Pupils advocated that this would address transport barriers, increasing accessibility and reducing dependence on parents/guardians.

#### Increased range of opportunities

Girls felt there were more activities on offer for boys and would like to see more choice of girls’ activities. The choice was restricted somewhat by facilities on offer within the area. Participants were encouraged to set up new activities, however, girls preferred to use pre-existing facilities.

Participants wanted access to bigger sports shops, which were unable to participate due to company restrictions allowing participation in nationwide schemes only. Smaller shops were available, but tended to be further from participants’ neighbourhoods. More participants may have used their vouchers for equipment had bigger, more accessible shops been available. The ability to use vouchers outside of the immediate area was recommended in order to further increase choice and opportunities for new activities.

#### Use during school holidays

With the scheme ending in July, recurring views suggested the scheme would be greatly missed during school holidays, as they would have more time for activity engagement. Furthermore, parents would have less money in July for them to spend on activities due to spending large amounts on new equipment for the impending school year.

### Teacher’s opinions

#### Increased opportunities

Teachers spoke highly of the scheme stating it provided pupils with opportunities they may not otherwise have had and encouraged adolescents to spend their leisure time more positively (Table 3).

**Table 3:** Qualitative data from teachers regarding positive aspects of the voucher scheme and recommendations for improvement

Positive aspects of the voucher scheme	
Increased range of opportunities	*"…I think that our kids have benefitted from it hugely because they have very limited extra income to do anything with and are very much led by their parents a lot of the time and a lot of the parents have quite a negative attitude towards exercise anyway and it’s broadened their horizons because it’s allowed them to go and try things that maybe they wouldn’t have had access to before because of financial constraints…" (T2)* *"…obviously it’s a good scheme because it provides the children with greater opportunities to do things outside of school and anything to encourage them to use their time positively has got to be good…" (T1)*
Positive restriction to physical activity or sport-related equipment	*"…it’s good that the vouchers can only be spent on the sport because even if the family’s more affluent there’s nothing to say they’d pass money onto the children who would then use it to buy activities in some way, they would possibly spend it on PlayStation games or mobile phones and stuff…" (T1)*
Engaged non-sporty individuals	*"…it's engaged a lot more of the girls, a lot of the girls that were really disaffected with PE before because of the more traditional sports that we often offer, have bothered to come down, to see us to talk to us about the options, chasing vouchers etcetera and that’s led to us having more engaging conversations which in turn has allowed them to feel a bit more relaxed down here and not so under pressure to perform, perform, perform all the time and it’s just more about the taking part” (T2)* *"… bridges have been built with certain girls who just disliked coming to PE in the past and now those bridges have been built and relationships have improved I think yeah, well hopefully, as far I’m concerned… it’s allowed them, a lot of them to take part more with a smile on their face and enjoy it more than they have done in the past…" (T2)* *"…Oh (name of child) went out and bought footballs and basketballs and she’s not a sporty person, to say the least. So yeah, I’d say there are some who’ve done things I wouldn’t have thought they would…" (T1)*
Recommendations for improvement	
Increase accessibility	*“…In a more affluent area, you’ve got two parents probably, they’ve got a car each and it’ll just happen and also from the cultural side of it, people from those areas probably themselves are taken places by their parents to do whatever activity they did and they’ll see it as an expectation for them to take their children, which you may not get up here…” (T1)* *“…Or you make the activity local to the children so most of our lot come from (deprived area) so you put stuff on at the Centre or the youth club up there so they can get to it…or putting coaches in because I mean every community’s got a bit of open land… Yeah, community, yeah, smaller community things…” (T1)*

#### Positive restriction to PA or sport-related equipment

The restriction of vouchers for PA was seen as an advantage as this prevented money being spent on other things, such as ‘Playstation’ games. Distributing vouchers directly to participants enabled them to be more involved as there was no need to negotiate money through parents, thereby removing a potential barrier.

#### Engaged non-sporty individuals

The Head of P.E. reported that the scheme enabled staff to build relationships with pupils who were usually disengaged with school-based PA. Pupils actively approached teaching staff to discuss the vouchers and where they could be used, leading to more pupils attending P.E. classes and willingly participating.

#### Recommendations

Teachers’ recommendations centred on increasing accessibility of existing activities, such as improved public transport to distant facilities or introduction of activities directly into communities, reducing the need for dependence on parents for transportation.

## Discussion

This study demonstrated that the ACTIVE scheme is feasible and strongly supported by pupils and teachers. Interestingly, teachers commented on increased engagement in P.E. from those usually disinterested. This may be due to some children avoiding sport that is competitive [26]; the vouchers counteracted this by empowering participants to choose activities they wanted to participate in, leading to an increased sense of involvement. This was significant as attitudes towards PA influence participation in adolescents [27, 28]. Indeed, whilst removal of cost was a large incentive to participate in PA, qualitative analysis showed that the scheme appeared to act through changing pre-conceived attitudes towards PA. Therefore, PA became a fun opportunity to socialise with friends, especially for girls, and the scheme allowed relationships with family and friends to develop through co-participation in activity. This is consistent with previous research reporting participation with friends promoted increased activity in young people [29, 30] and motivation to be physically active [31].

In our study, spontaneous, unplanned activities were favoured, such as ‘Laserzone’ or skateboarding; consistent with the behaviour of children from lower socioeconomic status [32]. This supports previous research suggesting adolescents see structured, timetabled activities as a barrier to PA [15].

Analysis of voucher usage revealed that boys were more likely to use vouchers for activity, and increased PA levels would most likely be the main contributor to increased fitness. Girls preferred to purchase equipment, such as roller blades and scooters. However, focus groups indicated that some lacked the ability to rollerblade, so were unlikely to increase their PA enough to significantly improve fitness. Competence and skill level have previously been reported as barriers to PA among teenage girls [8, 15]. Thus, future work should explore how to improve fundamental movement skills in order to enable greater use of sporting equipment, and further increase PA.

Qualitative responses demonstrated that improving activity levels is a complex, multi-faceted issue; the scheme addressed a number of issues but additional barriers to PA such as transport, time and variety remained. Recommendations included provision of an electronic card, expansion to neighbouring counties, and administration of vouchers in school holidays. Another major barrier to PA confirmed in previous work was transport to and from facilities [13]. In this study, both pupils and teachers recommended reducing public transport costs and increasing opportunities for free bus passes. Improving access to facilities through better transport would greatly enhance the success of future similar interventions. These recommendations provide useful insights into barriers experienced by adolescents, which are invaluable for ensuring their needs are met when developing future studies.

Early observations presented favourable changes in PA, SB and fitness in both boys and girls. However, these changes appeared to be more pronounced at the weekend. This is consistent with the qualitative reports of increased time for activity and increased voucher usage during non-school days. This is promising given the literature reports less activity during weekends [33]. Conversely, weekday data demonstrated increased time spent in SB. No explanation for this was presented qualitatively but it could be a compensatory result of undertaking more activity during the weekend [34]. However, a recent review has reported existing research regarding the presence of compensation as inconclusive [35].

An additional point to note is that although the school itself was classified as deprived (54 % FSM eligibility), not all pupils who participated were explicitly classified as deprived; a common limitation for school-based projects. Given the small sample size, it was encouraging to see such positive differences in behaviour. However, the next step is to examine effect size and uptake in a larger range of schools, including comparators, to examine who benefits most from vouchers and whether the collective distribution of vouchers amongst adolescents plays a role in engagement. Cost-effectiveness, to explore the cost-benefit for changes in physical activity, and long-term sustainability also warrant further exploration. Additionally, this study only examined PA, SB and fitness. However, when children choose activities such as weight lifting or yoga, it is likely that different aspects of fitness (not measured here) would improve. Therefore, further research is required to examine which other outcomes are affected when teenagers do activities of their choice rather than traditional sports.

## Conclusions

This study demonstrates that ACTIVE is a feasible approach to increasing PA and fitness amongst adolescents from a low socioeconomic background. This was achieved predominantly through changing attitudes towards PA and reducing cost barriers, allowing those from deprived backgrounds more opportunities to access activities. The feasibility of this approach on a larger scale, alongside a greater understanding of effectiveness, needs exploration.

## Abbreviations

*ACTIVE*, active children through incentive vouchers—evaluation; *BREQ-2*, behavioural regulation in exercise questionnaire; *COREQ*, consolidated criteria for reporting qualitative research; *CRT*, cooper run test; *FSM*, free school meals; *HBAI*, households below average income data; *LPA*, light physical activity; *MVPA*, moderate-to-vigorous physical activity; *P.E.*, physical education; *PA*, physical activity; *PAQ-A*, physical activity questionnaire for adolescents; *RE-AIM framework*, used to evaluate reach, effectiveness, adoption, implementation and maintenance of an intervention; *SB*, sedentary behaviour; *UK*, United Kingdom

## Additional file

Additional file 1: Evidence of effect. The data within Additional file 1 includes more details regarding the quantitative outcomes from the ACTIVE study such as physical activity, sedentary behaviour, fitness and motivation to exercise. (PDF 239 kb)
