# Supplementary material for: Active children through incentive vouchers – evaluation (ACTIVE): a mixed-method feasibility study
Source: BMC Public Health. 2016 Aug 26;16(1):890. doi: 10.1186/s12889-016-3381-6 (PMC5002124; doi:10.1186/s12889-016-3381-6)
Supplement: Additional file 1: — Evidence of effect. The data within Additional file 1 includes more details regarding the quantitative outcomes from the ACTIVE study such as physical activity, sedentary behaviour, fitness and motivation to exercise. (PDF 239 kb) [file 12889_2016_3381_MOESM1_ESM.pdf]

Additional file 1 - Evidence of effect

Quantitative outcomes

Physical activity (PA): Objectively measured using the GENEActiv © triaxial accelerometer (GENEActiv, Unilever Discover, Sharnbrook, Bedfordshire, UK). When worn on the left wrist, mainly classified as the non-dominant wrist, the GENEA accelerometer has demonstrated excellent criterion validity in children (r = 0.91) [1]. Accelerometers were programmed to collect data at 100 Hz and pupils were instructed to wear the monitor 24 hours per day, including whilst sleeping and during water-based activities. The physical activity questionnaire for adolescents (PAQ-A) was used to determine differences in self-reported activity and add context to the objective data [2]. The PAQ-A is a seven day recall PA questionnaire; it is a valid and reliable measure of PA that has been widely used in research [3].

Aerobic fitness: The 12 minute Cooper run test (CRT) is commonly used to estimate aerobic fitness among children and adolescents [4-6]. The CRT took place during scheduled Physical Education lessons in the school sports hall, whereby pupils were encouraged to walk/run as far as possible around a pre-measured course during a 12 minute time allocation.

Motivation to exercise: Motivation to exercise was assessed using the Behavioural Regulation in Exercise Questionnaire (BREQ-2) [7] which has shown validity and reliability within adolescent populations [8]. The BREQ-2 consists of 19 items, scored on a 5-point Likert scale, which measures the motivation to exercise ranging from amotivation (lack of motivation) through to intrinsic regulation (choosing freely to exercise because it is enjoyable).

Baseline PA (objectively-measured and self-reported) and aerobic fitness measures were obtained one month prior to the intervention, and were repeated during the fifth month of the intervention period and again after six months of follow-up post intervention. Motivation to exercise was measured mid-intervention and again at follow-up (Figure 1).

Data Analysis

Accelerometry data were downloaded and converted to 60-second epoch .csv files using GENEActiv PC software version 2.1. These 60-second epoch data files were entered into an open source Excel macro (version2; Activinsights Ltd.), designed and validated for 60-second epochs, in order to classify non-wear and sleep time, as described by Metcalf and colleagues [9]. KineSoft software (version 3.3.75; KineSoft, Loughborough, UK) was then used to produce PA intensities (min.day^{-1}) for each participant-day following procedures similar to those described elsewhere [10]. The widely used wear time criteria of 600 minutes on any three days was applied [11]. In addition, validated cut-points were used to classify sedentary behaviour, light physical activity (LPA) and moderate-to-vigorous physical activity (MVPA) [1].

Paired t-tests were conducted to investigate the differences from baseline to post-intervention and follow-up for PA and fitness and similarly for motivation to exercise at two measurement time points: mid-intervention and six-month follow-up. Paired t-tests were used instead of repeated measures ANOVA due to unequal numbers of observations between time-points, selection bias and loss of power if observations are required at all three time-points. Additionally, the assumption that compound variance would not differ in this instance could not be guaranteed. Preliminary analyses to ensure normal distribution of data were completed prior to all further analyses. Descriptive statistics (frequencies/percentages) were used to describe

levels of engagement with the scheme and to gain insight into voucher usage. STATA V.12.1 (STATA, Texas, USA) was used for all statistical analyses and statistical significance was set at $p<0.05$ throughout.

Physical Activity and Fitness

Objectively assessed PA showed a marginal increase in MVPA during the voucher scheme (Table 4). When stratified by day of the week, weekend MVPA showed a significant increase during intervention in both sexes (Table 5). Similarly, LPA increased marginally over the six month period (Table 4), though this was only shown to be significant during weekdays. These increases were not maintained when vouchers were removed. A significant decrease in sedentary time was reported overall during the intervention, though this was not sustained twelve months post-baseline.

Pupils classed as 'non-active' (MVPA<60min.day^{-1}) at baseline demonstrated an increase in number of minutes spent in MVPA, increasing from 37 ±14.8 to 53 ±14.2 minutes during the intervention (n=5, p=0.09). However, on average, children classified as ‘non-active’ reduced their MVPA marginally so twelve month follow-up values were comparable to baseline, from 42.8 ±15.6 to 36.8 ±13.6, respectively (n=7, p=0.3). The same trends were observed in 'active' pupils (MVPA≥60min.day^{-1}), though did not reach significance; increases at post-intervention (difference +5mins (95%CI: -21.0 to 10.4, n=21, p=0.49) and decreases to follow-up (difference -12mins (95%CI: -4.3 to 29.8). Self-reported habitual PA (PAQ-A) did not change throughout all three time points.

The intervention was associated with a significant improvement in fitness at post-intervention (Table 4). Stratification by sex showed boys’ fitness improved significantly, whereas girls’ improved only marginally. These improvements were not

sustained for boys at the twelve month follow-up and returned to baseline fitness levels after the vouchers were stopped. However, girls' fitness remained comparable to that found directly after the intervention.

The fitter, more active participants attended the baseline tests (n=87) and a greater proportion of those less fit attended at the end of the intervention (n=95). Fitness at baseline and post-intervention in the unfit pupils (defined as <1600m (girls) and <2200m (boys)[12]) was 1561.5m & 1687.4m respectively (difference: 125.9m (95%CI: 212.7 to 39), n = 54, p=0.005) and at twelve months follow-up 1637.2m (72.6m (95%CI: -32.4 to 177, n = 43, p=0.17). Thus, in unfit participants there was a significant improvement in fitness from baseline during the intervention and a trend for fitness to be maintained even after the intervention had finished.

Factors affecting voucher usage

The amount of time spent in sedentary pursuits at baseline (classified as sedentary minutes per day>median (667.4 min.day^{-1})) had no effect on voucher usage. However, being classified as 'non-active' at baseline (MVPA<60min.day^{-1}) was significantly associated with higher voucher use than 'active' pupils (21 ± 9.6 & 12 ± 10.8 vouchers, respectively, p=0.007). Pupils who were of average fitness or above at baseline (CRT score >1600m (girls) and CRT score >2200m (boys) [12], n=61) showed no difference in voucher usage compared to those below average fitness (n=54), (13 ±10.7 & 13 ±11.5 vouchers used, respectively, p=0.9). No significant differences with sex or free school meal eligibility were observed.

Voucher usage was also significantly associated with motivation scores, suggesting that those who were more motivated used more vouchers (regression coefficient 0.71 (p=0.001) using motivation as a continuous variable; most motivated 50% used 15 vouchers on average; less motivated 50% used 10 vouchers).

References

1. Phillips L, Parfitt G, Rowlands AV: Calibration of the GENEA accelerometer for assessment of physical activity intensity in children. *Journal of Science and Medicine in Sport* 2013, 16(2):124–128.
2. Haskell WL: Physical activity by self-report: a brief history and future issues. *Journal of Physical Activity and Health* 2012, 9(Suppl 1):S5-S10.
3. Kowalski KC, Crocker PRE, Kowalski NP: Convergent validity of the Physical Activity Questionnaire for Adolescents. *Pediatric Exercise Science* 1997, 9:342-352.
4. Cooper KH: A means of assessing maximal oxygen intake. Correlation between field and treadmill testing. *JAMA* 1968, 203(3):201-204.
5. Drinkard B, McDuffie J, McCann S, Uwaifo GI, Nicholson J, Yanovski JA: Relationships between walk/run performance and cardiorespiratory fitness in adolescents who are overweight. *Phys Ther* 2001, 81:1889–1896.
6. Calders P, Deforche B, Verschelde S, Bouckaert J, Chevalier F, Bassle E, Tanghe A, De Bode P, Franckx H: Predictors of 6-minute walk test and 12-minute walk/run test in obese children and adolescents. *European Journal of Pediatrics* 2008, 167:563–568.
7. Markland D, Tobin V: A modification to the behavioural regulation in exercise questionnaire to include an assessment of amotivation. *Journal of Sport and Exercise Psychology* 2004, 26(2):191-196.
8. Gillison FB, Standage M: An examination of the psychometric properties of the behavioural regulation in exercise questionnaire-2 (BREQ-2) within an adolescent population. *Br Psychol Soc Proc* 2005, 13.
9. Metcalf KM, Singhvi A, Tsalikian E, Tansey MJ, Zimmerman MB, Esliger DW, Janz KF: Effects of Moderate-to-Vigorous Intensity Physical Activity on Overnight and Next-Day Hypoglycemia in Active Adolescents With Type 1 Diabetes. *Diabetes Care* 2014, 37(5):1272-1278.
10. Esliger DW, Copeland JL, Barnes JD, Tremblay MS: Standardizing and optimizing the use of accelerometer data for free-living physical activity monitoring. *Journal of Physical Activity and Health* 2005., 3:366-383.
11. Mattocks C, Ness AR, Leary SD, Tilling K, Blair SN, Shield J, Deere K, Saunders J, Krikby J, Smith GD: Use of accelerometers in a large field-based study of children: protocols, design issues, and effects on precision. *Journal of Physical Activity and Health* 2008, 5(Supplement 1):S98.
12. Mackenzie B: Cooper VO2 max Test: http://www.brianmac.co.uk/gentest.htm (Access date 23/03/2016). 1997.

Table 4 Changes in fitness, physical activity, sedentary behaviour and motivation between baseline, post-intervention and follow-up

		Total	***Girls***	***Boys***
Cooper run		**n= 75**	n= 36	**n=39**
(Matched)	Baseline	**1730.1 (406.0)**	1517.8 (259.0)	**1926.2 (420.7)**
(Distance, m)	Post-intervention	**1828.3 (534.0)**	1540.0 (302.6)	**2094.4 (565.3)**
	Difference	**98.1* (344.4)**	22.2 (286.6)	**168.2** (380.5)**
	(95%CI)	**(18.9 to 177.4)**	(-74.8 to 119.2)	**(44.9 to 291.5)**
		n=63	n=32	n=31
	Baseline	1752.4 (411.6)	1552.5 (281.5)	1958.7 (426.3)
	Follow-up	1746.0 (445.5)	1567.5 (263.3)	1930.3 (519.2)
	Difference	-6.4 (348.2)	15.0 (307.8)	-28.4 (389.5)
	(95%CI)	(-94.0 to 81.3)	(-96.0 to 126.0)	(-171.3 to 114.5)
MVPA		n=26	n=14	n=12
(min.day^{-1})	Baseline	105.4 (49.8)	89.6 (54.5)	123.8 (37.8)
	Post-intervention	112.8 (48.0)	98.0 (44.6)	130.0 (47.9)
	Difference	7.4 (31.9)	8.4 (39.1)	6.2 (22.3)
	(95%CI)	(-5.4 to 20.3)	(-14.2 to 31.0)	(-7.9 to 20.5)
		n=31	n=16	n=15
	Baseline	99.5 (46.9)	87.0 (51.4)	112.9 (39.0)
	Follow-up	88.3 (47.1)	71.3 (45.3)	106.5 (43.3)
	Difference	-11.2 (36.3)	-15.7 (41.7)	-6.4 (30.1)
	(95%CI)	(-24.5 to 2.1)	(-37.9 to 6.5)	(-23.1 to 10.3)
LPA		n=26	n=14	n=12
(min.day^{-1})	Baseline	264.9 (57.0)	275.6 (53.7)	252.4 (60.4)
	Post-intervention	295.5 (110.3)	307.2 (85.7)	281.9 (136.4)
	Difference	30.7 (110.9)	31.6 (87.5)	29.6 (137.5)
	(95%CI)	(-14.1 to 75.4)	(-18.9 to 82.1)	(-57.8 to 116.9)
		n=31	n=16	n=15
	Baseline	253.4 (51.2)	273.5 (48.2)	231.8 (46.6)
	Follow-up	225.5 (76.2)	238.0 (91.9)	212.1 (55.1)
	Difference	-27.9 (79.4)	-35.5 (97.7)	-19.7 (56.3)
	(95%CI)	(-57.0 to 1.3)	(-87.6 to 16.5)	(-50.9 to 11.4)
Sedentary		**n=26**	n=14	n=12
(min.day^{-1})	Baseline	**687.3 (78.5)**	696.6 (77.4)	676.4 (81.8)
	Post-intervention	**622.5 (149.9)**	629.7 (149.2)	614.1 (156.9)
	Difference	**-64.8* (130.7)**	-66.9 (150.0)	-62.3 (110.6)
	(95%CI)	**(-117.6 to -12.0)**	(-153.5 to 19.7)	(-132.6 to 8.0)
		n=31	n=16	n=15
	Baseline	687.9 (90.9)	685.2 (76.5)	690.7 (106.8)
	Follow-up	687.4 (117.8)	667.0 (132.4)	709.2 (99.8)
	Difference	-0.5 (112.0)	-18.2 (125.2)	18.5 (96.6)
	(95%CI)	(-41.6 to 40.5)	(-84.9 to 48.5)	(-35.1 to 71.9)
PAQ		n = 75	n = 34	n = 41
	Baseline	2.6 (0.9)	2.4 (0.7)	2.8 (1.0)
	Post-intervention	2.7 (0.9)	2.3 (0.7)	2.9 (1.0)
	Difference	0.1 (0.8)	-0.1 (0.7)	0.2 (0.9)
	(95%CI)	(-0.1 to 0.3)	(-0.3 to 0.2)	(-0.1 to 0.5)
		n=61	n=32	n=29
	Baseline	2.6 (0.8)	2.5 (0.8)	2.7 (0.9)
	Follow-up	2.5 (0.9)	2.4 (0.8)	2.6 (0.9)

	Difference	-0.1 (0.7)	-0.1 (0.8)	-0.1 (0.6)
	(95%CI)	(-0.3 to 0.1)	(-0.4 to 0.2)	(-0.3 to 0.1)
Motivation		**n = 78**	n = 43	**n = 35**
	Mid-intervention	**6.8 (6.0)**	6.0 (5.3)	**7.9 (6.7)**
	Follow-up	**5.1 (6.7)**	5.1 (6.1)	**5.0 (7.6)**
	Difference	**-1.7* (6.1)**	-0.9 (6.7)	**-2.8** (5.1)**
	(95%CI)	**(-3.1 to -0.4)**	(-2.9 to 1.2)	**(-4.6 to -1.0)**

Data represented as Mean (SD), unless otherwise stated. Mid-intervention refers to three months post-baseline, post-intervention refers to five months post-baseline and follow-up refers to twelve months post-baseline. Bold = achieves significance *(p<0.05), **(p<0.01), ***(p<0.001)

Table 5 Changes in sedentary behaviour, LPA and MVPA stratified by weekday or weekend between baseline, post-intervention and follow-up

		Total	***Girls***	***Boys***
**Weekday**		n=26	n=14	n=12
**MVPA**	Baseline	118.3 (54.0)	102.2 (60.4)	137.1 (40.1)
**(*min.day^{-1}*)**	Post-intervention	116.6 (52.8)	98.8 (49.7)	137.4 (50.5)
	Difference	-1.7 (33.7)	-3.4 (39.9)	0.3 (26.3)
	(95%CI)	(-15.3 to 11.9)	(-26.5 to 19.6)	(-16.3 to 17.1)
		n=30	n=15	n=15
	Baseline	116.7 (56.9)	98.7 (58.9)	134.7 (50.6)
	Follow-up	101.8 (51.4)	82.9 (51.2)	120.7 (45.7)
	Difference	-14.9 (41.6)	-15.8 (45.7)	-14.0 (38.5)
	(95%CI)	(-30.4 to 0.6)	(-41.1 to 9.6)	(-35.3 to 7.3)
**Weekend**		**n=24**	**n=13**	n=11
**MVPA**	Baseline	**76.5 (52.2)**	**61.5 (55.7)**	94.1 (43.7)
**(*min.day^{-1}*)**	Post-intervention	**116.9 (80.6)**	**95.8 (46.7)**	141.8 (105.2)
	Difference	**40.4** (62.0)**	**34.3* (50.3)**	47.7 (75.4)
	(95%CI)	**(14.3 to 66.6)**	**(3.9 to 64.7)**	(-3.0 to 98.3)
		n=28	n=15	n=13
	Baseline	57.5 (34.9)	48.8 (27.6)	67.6 (40.5)
	Follow-up	54.8 (42.4)	42.2 (35.0)	69.5 (46.8)
	Difference	-2.7 (41.2)	-6.7 (40.6)	1.9 (43.1)
	(95%CI)	(-18.7 to 13.3)	(-29.1 to 15.8)	(-24.1 to 27.9)
**Weekday**		**n=26**	n=14	n=12
**LPA**	Baseline	**215.8 (47.8)**	223.0 (47.5)	207.4 (48.7)
**(*min.day^{-1}*)**	Post-intervention	**263.4 (93.5)**	271.3 (106.8)	254.1 (78.9)
	Difference	**47.6** (85.4)**	48.2 (95.8)	46.8 (75.7)
	(95%CI)	**(13.0 to 82.1)**	(-7.1 to 103.6)	(-1.3 to 94.9)
		n=30	n=15	n=15
	Baseline	209.1 (44.2)	222.2 (45.3)	196.1 (40.4)
	Follow-up	214.6 (78.1)	226.8 (94.8)	202.4 (57.8)
	Difference	5.5 (78.4)	4.7 (93.8)	6.3 (62.8)
	(95%CI)	(-23.8 to 34.8)	(-47.3 to 56.6)	(-28.5 to 41.1)
**Weekend**		n=24	n=13	n=11
**LPA**	Baseline	250.1 (71.0)	255.9 (58.0)	243.3 (86.4)
**(*min.day^{-1}*)**	Post-intervention	299.7 (156.9)	291.1 (72.7)	309.9 (223.8)
	Difference	49.6 (147.9)	35.2 (81.9)	66.6 (204.1)
	(95%CI)	(-12.8 to 112.1)	(-14.3 to 84.7)	(-70.6 to 203.7)
		n=28	n=15	n=13
	Baseline	214.6 (80.6)	239.8 (77.1)	185.6 (77.2)
	Follow-up	178.7 (94.3)	201.1 (115.9)	152.8 (54.7)
	Difference	-36.0 (98.4)	-38.7 (118.0)	-32.8 (74.4)
	(95%CI)	(-74.1 to 2.2)	(-104.0 to 26.6)	(-77.7 to 12.1)
**Weekday**		**n=26**	**n=14**	**n=12**
**Sedentary**	Baseline	**572.0 (85.9)**	**598.3 (87.2)**	**541.4 (76.6)**
**(*min.day^{-1}*)**	Post-intervention	**657.4 (166.5)**	**677.2 (165.4)**	**634.4 (172.0)**
	Difference	**85.4** (128.4)**	**78.9* (127.9**	**93.0* (134.2)**
	(95%CI)	**(33.5 to 137.2)**	**(5.0 to 152.8)**	**(7.7 to 178.3)**
		**n=30**	n=15	**n=15**
	Baseline	**578.1 (99.3)**	606.9 (72.0)	**549.4 (116.0)**
	Follow-up	**675.9 (118.7)**	666.2 (133.3)	**685.7 (105.8)**
	Difference	**97.8*** (123.6)**	59.3 (122.3)	**136.3*** (116.1)**
	(95%CI)	**(51.7 to 143.9)**	(-8.5 to 127.1)	**(72.0 to 200.6)**
**Weekend**		**n=24**	**n=13**	**n=11**
**Sedentary**	Baseline	**961.7 (211.0)**	**920.7 (227.8)**	**1010.0 (188.0)**
**(*min.day^{-1}*)**	Post-intervention	**560.9 (163.3)**	**520.3 (140.1)**	**608.8 (181.9)**
	Difference	**-400.8*** (237.1)**	**-400.4*** (231.7)**	**-401.2*** (254.7)**

	(95%CI)	**(-500.9 to -300.7)**	**(-540.4 to -260.4)**	**(-572.3 to -230.1)**
		**n=28**	**n=15**	**n=13**
	Baseline	**985.3 (206.6)**	**903.9 (185.0)**	**1079.2 (195.7)**
	Follow-up	**731.6 (199.9)**	**673.0 (196.3)**	**799.3 (188.9)**
	Difference	**-253.7*** (295.5)**	**-231.0* (308.1)**	**-279.9** (290.4)**
	(95%CI)	**(-368.3 to -139.1)**	**(-401.6 to -60.3)**	**(-455.4 to -104.4)**

Data represented as Mean (SD), unless otherwise stated. Post-intervention refers to five months post-baseline and follow-up refers to twelve months post-baseline. Bold = achieves significance *(p<0.05), **(p<0.01), ***(p<0.001)
